# Implementation barriers and facilitators of an integrated multidisciplinary lifestyle enhancing treatment for inpatients with severe mental illness: the MULTI study IV

**DOI:** 10.1186/s12913-019-4608-x

**Published:** 2019-10-22

**Authors:** Jeroen Deenik, Diederik E. Tenback, Erwin C. P. M. Tak, Olivier A. Blanson Henkemans, Simon Rosenbaum, Ingrid J. M. Hendriksen, Peter N. van Harten

**Affiliations:** 1 GGz Centraal, Utrechtseweg 266, 3818EW Amersfoort, the Netherlands; 2 School for Mental Health and Neuroscience, Maastricht University, PO Box 616, 5200MD Maastricht, the Netherlands; 3 Tak Advies en Onderzoek, Hooigracht 38/K, 2312KV Leiden, the Netherlands; 4 TNO Leiden, Schipholweg 77, 2316ZL Leiden, the Netherlands; 5 School of Psychiatry, University of New South Wales, Hospital Road, Randwick NSW, Sydney, 2031 Australia; 6 Black Dog Institute, Prince of Wales Hospital, Hospital Road, Randwick NSW, Sydney, 2031 Australia; 7 LivIng Active, Middenduinerweg 67, 2082LC Santpoort-Zuid, the Netherlands

**Keywords:** Physical activity, Severe mental illness, Schizophrenia, Lifestyle, Implementation

## Abstract

**Background:**

Despite an increase in studies showing the efficacy of lifestyle interventions in improving the poor health outcomes for people with severe mental illness (SMI), routine implementation remains ad hoc. Recently, a multidisciplinary lifestyle enhancing treatment for inpatients with SMI (MULTI) was implemented as part of routine care at a long-term inpatient facility in the Netherlands, resulting in significant health improvements after 18 months. The current study aimed to identify barriers and facilitators of its implementation.

**Methods:**

Determinants associated with the implementation of MULTI, related to the innovation, the users (patients, the healthcare professionals (HCPs)), and the organisational context, were assessed at the three wards that delivered MULTI. The evidence-based Measurement Instrument for Determinants of Innovations was used to assess determinants (29 items), each measured through a 5-point Likert scale and additional open-ended questions. We considered determinants to which ≥20% of the HCPs or patients responded negatively (“totally disagree/disagree”, score < 3) as barriers and to which ≥80% of HCPs or patients responded positively (“agree/totally agree”, score > 3) as facilitators. We included responses to open-ended questions if the topic was mentioned by ≥2 HCPs or patients. In total 50 HCPs (online questionnaire) and 46 patients (semi-structured interview) were invited to participate in the study.

**Results:**

Participating HCPs (*n* = 42) mentioned organisational factors as the strongest barriers (e.g. organisational changes and financial resources). Patients (*n* = 33) mentioned the complexity of participating in MULTI as the main barrier, which could partly be due to organisational factors (e.g. lack of time for nurses to improve tailoring). The implementation was facilitated by positive attitudes of HCPs and patients towards MULTI, including their own role in it. Open responses of HCPs and patients showed strong commitment, collaboration and ownership towards MULTI.

**Conclusions:**

This is the first study analysing the implementation of a pragmatic lifestyle intervention targeting SMI inpatients in routine clinical care. Positive attitudes of both HCPs and patients towards such an approach facilitated the implementation of MULTI. We suggest that strategies addressing organisational implementation barriers are needed to further improve and maintain MULTI, to succeed in achieving positive health-related outcomes in inpatients with SMI.

## Background

It is well established that the implementation of lifestyle interventions in people with severe mental illness (SMI), such as psychotic disorders, in psychiatry is a major challenge. The urgency to improve physical health is stressed by the substantially reduced life expectancy of 7–20 years compared to the general population [1–3]. This is largely caused by poor cardiovascular health [2, 4–7], in which modifiable lifestyle factors such as lack of physical activity [8–12], smoking [13] and poor nutrition [14] play a major role. In recent decades, there has been an increase in research aimed at addressing this health disparity by improving this lifestyle. Smoking-cessation interventions have been shown to be effective, also in the longer term [15, 16]. Regarding the efficacy of increasing physical activity, many systematic reviews and meta-analyses showed improvements on cardiometabolic health, aerobic capacity, global functioning, psychiatric symptoms, quality of life and cognitive functioning in patients with SMI, with most efficacious interventions delivered by qualified exercise professionals and executed at sufficient levels of intensity [17–20]. Interventions that (partly) addressed dietary risks yielded improvements in cardiometabolic risk factors, with larger effect sizes for interventions delivered by qualified professionals, as well [21, 22]. Although these studies were vital to show that lifestyle interventions work (i.e. efficacy), the evidence to support the long-term sustainability of lifestyle interventions for patients with SMI is currently limited [18, 21, 23]. The gap between the increase in evidence and policies regarding lifestyle interventions for people with SMI and changes in routine care has been stressed several times, calling for action [24–28]. There is a need for more research studying the effectiveness (i.e. how patients with SMI can include such changes in their daily lives) [21, 29–31] and implementation of lifestyle interventions in real-world settings [31] to further close this gap between research and practice. The challenge of implementing lifestyle interventions in patients with SMI was well illustrated in a pilot study, where patients were offered free access to fitness facilities, and 90% of the patients dropped out after 6 months [32]. Also, in a recent trial in SMI inpatients [33, 34], no long-term effects of a lifestyle intervention were found, and the authors hypothesized that implementation problems (partly) contributed to the null finding. Even more examples of negative findings in clinical practice likely exist, however, failed implementation studies are less likely to be published [35].

Understanding implementation barriers and facilitators can contribute to the interpretation of the results of interventions and to devise strategies to enhance the integration of research findings into routine clinical care [31, 36, 37]. They manifest themselves on levels of the intervention, the individual (patient), provider (healthcare professionals; HCPs), organisation, and community/system [38], although the latter two largely overlap in inpatient facilities. A previous study showed that the perceived benefits which may motivate patients with SMI to be physically active (such as mood improvement, stress reduction and improvement in physical health) were in contrast with perceived barriers (e.g. low mood and motivation, physical comorbidities, stress and side effects of medication) [39]. In addition, HCPs reported a lack of motivation in patients as the main obstacle to increasing physical activity [40–43]. Perceived barriers by HCPs were related to lack of time, support or training, competing work demands and organisational issues, e.g. prioritisation and lack of management support [43–46]. However, studies evaluating the implementation of lifestyle interventions in ‘real-world’ settings are scarce. Moreover, previous research has mainly focused on the individual perspective (both patients and HCPs), whereas there is a need for insight into (modifiable) barriers and facilitators at the organisational/environmental level as well [29, 38, 47, 48].

Recently, a multidisciplinary lifestyle enhancing treatment for inpatients with SMI (MULTI) was introduced in the Netherlands. A team of psychiatrists, nurses, activity coordinators, team leaders and a dietitian implemented MULTI within daily treatment with the purpose of achieving overall lifestyle change, focusing on decreasing sedentary behaviour, increasing physical activity and improving dietary habits. Through such an approach, MULTI aligns with the recommendations of recent studies that advocate for a multidisciplinary and holistic approach, supported by peers and qualified HCPs, including personalisation/tailoring, the use of multiple components and an organisational culture change [22, 43, 49–59]. Previously, a pragmatic evaluation of MULTI in the real-world setting after 18 months showed significant improvements in physical activity, metabolic health and psychosocial functioning [60, 61] and a decreased use of psychotropic medication [62], compared to treatment as usual. This was the first study to demonstrate such comprehensive long-term improvements in the inpatient population. The lack of improvements in physical activity and metabolic health in the treatment as usual group during these 18 months reinforces the need for systemic change within routine clinical care [60].

Nonetheless, also within the inpatient setting – in which there is less evidence regarding lifestyle interventions compared to outpatient settings [52, 63, 64] – the main challenge after studying the effectiveness of interventions targeting lifestyle lies in the implementation and maintenance of lifestyle interventions. Therefore, in addition to the previous evaluation of health-related outcomes, this study aimed to identify barriers to and facilitators of the implementation of MULTI. The results will enable us to better interpret previously studied outcomes of MULTI and devise strategies for future implementation of lifestyle interventions for this vulnerable population.

## Methods

### Study design

The current study complements a previous pragmatic evaluation of health-related outcomes 18 months after the start of MULTI (February 2014) as described elsewhere [60, 61]. After finishing data collection for this evaluation (December 2015), patients and HCPs involved in MULTI were invited to participate in the present study. In this study, we took multiple levels of implementation into account (i.e. the innovation itself, the users and the organisation), according to the framework as proposed by Fleuren et al. [65] based on a pooled analysis of studies and a Delphi study among – and consultation of – implementation experts. To identify implementation barriers and facilitators of MULTI on these different levels among HCPs (psychiatrists, nurses, activity coordinators, team leaders and a dietitian), they were invited by email to participate. They completed the questionnaire as an online survey using a unique link, to assess barriers and facilitators of implementing MULTI. Three reminder e-mails were sent at two-week intervals to all non-responders. Taking psychopathology and impaired cognitive abilities of patients into account, a trained research assistant conducted the questionnaire as a semi-structured interview to identify implementation barriers and facilitators of MULTI among patients. The Medical Ethical Committee of the Isala Academy approved the protocol (case 14.0678). All subjects gave (digital) written informed consent in accordance with the Declaration of Helsinki.

### Study population

This study was conducted at three long-stay wards (≥1 year) within a psychiatric hospital of GGz Centraal (the Netherlands). Patients and HCPs were included if they were treated or worked at the wards where MULTI was delivered (*N* = 65 and *N* = 56, respectively). Patients were excluded if they did not understand the content of MULTI, in consultation with their attending psychiatrist, and HCPs were excluded if they did not actively work with MULTI.

### MULTI

The purpose of MULTI was a holistic lifestyle change with a focus on decreasing sedentary behaviour, increasing physical activity and improving dietary habits among long-term inpatients with SMI. The treatment method was based on improving the daily structure, by starting each day with getting up on time, having three joint meals per day and participation in an active day program. The latter consisted of sports-related activities (e.g. walking, running, yoga, biking, indoor team sports), work-related activities (e.g. gardening and working in services within the hospital), psycho-education (e.g. about side effects of medication, dietary habits) and daily living skills training (e.g. shopping, cooking). Additionally, existing policies were critically reviewed and adjusted if necessary (e.g. limiting the use of personal transport by patients for trips within walking distance around the hospital area). Based on heterogeneity in patients’ illness severity, capabilities and interests, the content and intensity of the day-to-day program were tailored to the particular ward and individual patients to establish sustainable change. Therefore, the actual frequency, intensity, kind of activities and format (e.g. group or alone) could vary between patients and wards. However, it was intended that all patients were doing some of the activities in the morning and afternoon, to prevent prolonged periods lying in bed or sitting at the ward. Also, the participation of nurses in the day-to-day program was a core element.

MULTI was based on a ‘change from within’ principle, meaning that it was developed by current staff using current resources within routine clinical care. It was supervised and disseminated per ward by the head practitioner (a psychiatrist) as an innovative treatment method. Multidisciplinary work sessions led to detailed plans of the day-to-day programs which were shared by and between the different teams and discussed, thus leading to maximum participation and engagement needed to achieve culture change. Staff received support by the psychiatrists (psycho-education), activity coordinators and the dietitian. Adherence to and compliance with the treatment was discussed in the weekly multidisciplinary consultation. If a patient could not sufficiently participate in the day-to-day program (e.g. had problems getting out of bed or had low attendance during the selected activities), it was agreed to provide extra support, using motivational counselling by their mentor (one of the nurses) or psychiatrist and by consulting an activity coordinator or dietitian if needed.

### Measurement

To identify implementation barriers and facilitators of MULTI, we used the evidence-based Measurement Instrument for Determinants of Innovations (MIDI) [65, 66]. It was primarily designed to determine barriers and facilitators of implementation, to choose appropriate implementation strategies. In this study, it was used while running MULTI to learn from the experiences for future implementation and better interpretation of health outcome results. The MIDI comprises of four subscales, measuring determinants for implementation related to the intervention itself (7 items), the user (11 items), the organisation (10 items) and the socio-political context (1 item). Although in the original development of MIDI the user was seen as the main provider of the innovation (the HCPs), we considered the patients as users as well, due to their significant role in MULTI. The 29 items are scored using a five-point scale (totally disagree – totally agree). In accordance with the MIDI instruction guide [67] and based on input from clinical practice and testing the content and usability of the questionnaire with two HCPs, we made some adjustments to tailor the questionnaire to MULTI and its context. To gain more detailed insight into the implementation determinants in both HCPs and patients, we split *personal benefits/disadvantages* (item 8) into two separate items. To measure personal benefits and disadvantages and potential organisational changes affecting implementation, we included predefined answers based on the feedback of the HCPs who tested the questionnaire. For outcome expectations (both importance and probability), social support, subjective norm (both normative beliefs and motivation to comply) and self-efficacy, we included predefined answers based on the design of MULTI, supplemented by answers based on feedback while testing the questionnaire. Lastly, we did not assess the subscale ‘socio-political context’, as we assumed legislation and regulations to be the same at all wards of our hospital [65]. Additional file 1: Table S1 includes the final questionnaire used to assess the implementation determinants in the HCPs. For patients, we limited the number of items of this questionnaire to those that were relevant from the patients’ perspective, to increase the feasibility and prevent unnecessary burden. Therefore, we excluded *procedural clarity* (item 1), *correctness* (item 2), *self-efficacy* (item 17) and *knowledge* (item 18), as (evidence for) the description and use of MULTI was focused on HCPs as its main ‘implementers’. Also, the subscale regarding implementation determinants related to the organisation was excluded, as patients are not involved in the organisational structure and regulations. The aforementioned predefined answers were reduced as well and tailored to relevant topics from the patients’ perspective, based on consultation with patients and nurses and pilot testing in two interviews. In Additional file 2: Table S2 the final questionnaire used to assess implementation determinants in patients is shown. In addition, both HCPs and patients were asked to mention the implementation barriers and facilitators that they experienced which were not specifically addressed in MIDI, using open-ended questions.

### Statistical analysis

We used the Statistical Package for the Social Sciences (SPSS) version 23.0 for all analyses. Independent-samples t-tests and chi-square test were used to analyse potential differences between participating patients and non-participants (excluded/dropout) in gender, age, diagnosis and illness severity at the start of implementation. Such analyses were not possible for HCPs, as only HCPs that completed the questionnaire provided consent to participate.

For the evaluation of the MIDI questionnaire items, we used means and standard deviations for each implementation determinant, as well as the score per subscale, after recoding negatively stated items. Consistent with recent studies [68, 69] and in consultation with Dr. Fleuren (developer MIDI), we decided that items to which ≥20% of the HCPs and patients responded negatively (corresponding to “totally disagree/disagree”, score < 3) were considered barriers and those to which ≥80% of HCPs and patients responded positively (corresponding “agree/totally agree”, score > 3) were considered facilitators in the implementation of MULTI. Due to differences between scale ranges, the item *descriptive norm* (item 15, scored 1–7) and *awareness of the content of the treatment* (item 19, scored 1–4) were excluded in calculating mean subscale scores. To identify barriers and facilitators, *descriptive norm* was categorised as negative (score 1–3), neutral (score 4) and positive (score 5–7) and *awareness of the content of the treatment* as negative (score 1–2) or positive (score 3–4). To code the open-ended questions with regard to barriers and facilitators we used an inductive approach to determine topics and included the topics if mentioned by at least two HCPs or patients.

## Results

**Fig. 1:**
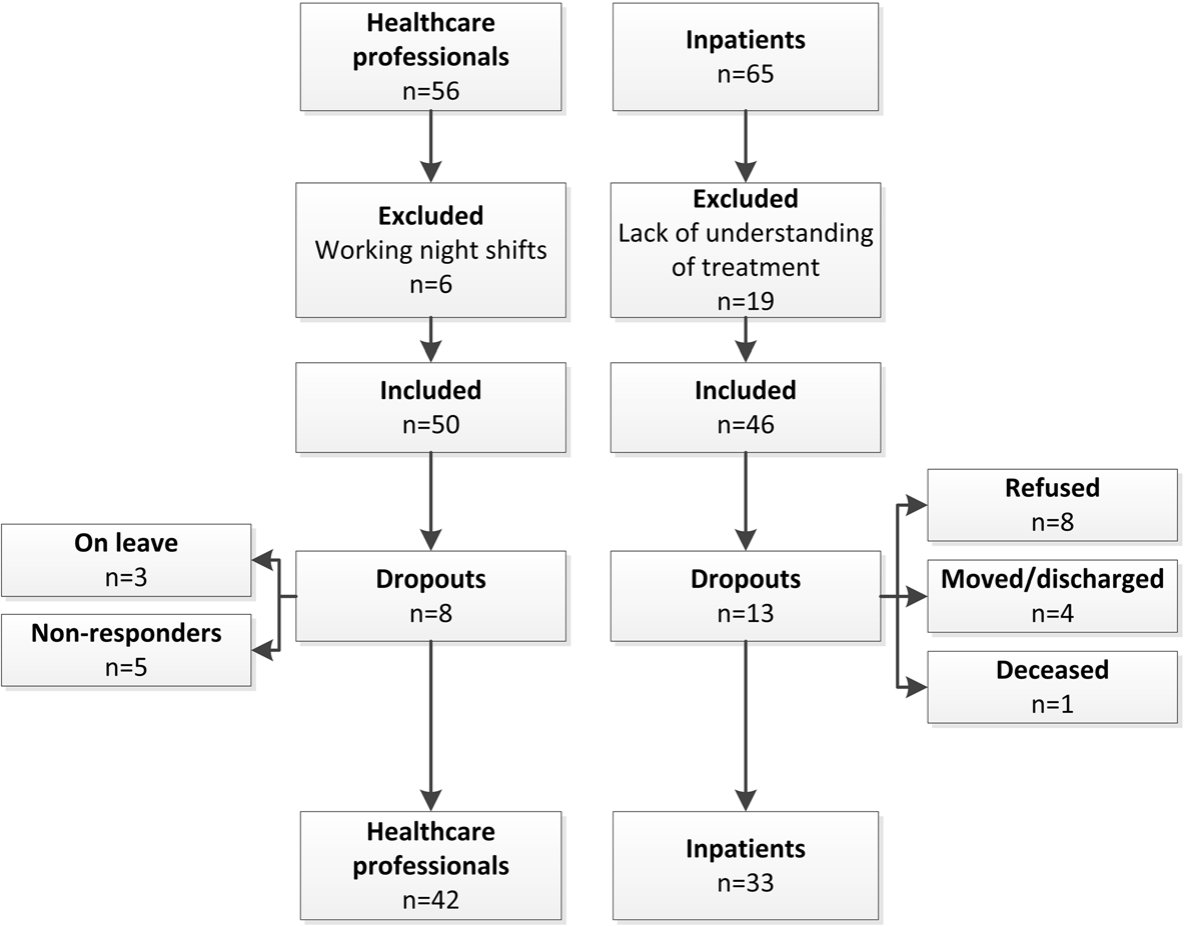
Flowchart of participants

Of the 50 eligible HCPs, 42 (84%) completed the questionnaire (see Fig. 1). All disciplines involved were represented. Activity coordinators represented both HCPs who focused on physical activity (*n* = 2) as well as HCPs who focused on other lifestyle training (*n* = 3, e.g. cooking). Compared to patients who were excluded (*n* = 19) or dropped out (*n* = 13), patients who completed the questionnaire (*n* = 33) were younger (*M* = − 4.90, 95% CI = − 9.19 to − 0.61), had lower illness severity (*M* = − 1.08, 95% CI = − 1.62 to − 0.53) and were hospitalised for fewer years (*M* = − 5.55, 95% CI = − 10.81 to − 0.28). Participating patients were mainly diagnosed with schizophrenia or related psychotic disorders (see Table 1). The internal consistency of the final questionnaire was fair to excellent for HCPs (intervention subscale: *α* = .84; user subscale: *α* = .93; organisation subscale: *α* = .74). In patients, this was poor for the intervention subscale (*α* = .48) and good for the user subscale (*α* = .86).

**Table 1:** Characteristics of patients and healthcare professionals (HCPs)

Outcome (scale)	HCPs (*N* = 42)	Patients (*N* = 33)
Sex, *n* (%) female	29 (69.0)	11 (33.3)
Age, years, mean (*SD*)	44.3 (12.7)	51.7 (8.7)
Patients’ illness characteristics		
Diagnosis schizophrenia or other psychotic disorder, *n* (%)		30 (90.9)
Non-psychotic disorder, *n* (%)		3^a^ (9.1)
Illness severity, CGI-S scale 1–7, mean (*SD*)		4.4 (1.1)
Years of hospitalisation, mean (*SD*)		11.6 (9.4)
HCPs disciplines, *n* (%)		
Nurse	26 (61.9)	
Nurse trainee	4 (9.5)	
Nurse practitioner	1 (2.4)	
Team leader	3 (7.1)	
Psychiatrist	2 (4.8)	
Activity coordinator	5 (11.9)	
Dietitian	1 (2.4)	

*Note.* CGI-S: Clinical Global Impression – Severity scale

^a^mood disorder (*n* = 1), a pervasive disorder not otherwise specified (*n* = 1) and an anxiety disorder (*n* = 1)

### Barriers

As shown in Table 2, HCPs only identified the completeness of MULTI (whether it provides all the information and materials needed to work with it properly) as a barrier regarding the intervention itself (21% responded negatively). Patients identified complexity (complicated to participate in MULTI; 67% responded negatively) and relevance (30% responded negatively) as barriers.

**Table 2:** Determinants associated with MULTI, HCPs/patients and organisation, including percentages of negative, neutral and positive responses

Determinants	HCPs (*N* = 42)					Patients (*N* = 33)				
	M	SD	% neg.	% neu.	% pos.	M	SD	% neg.	% neu.	% pos.
Determinants of MULTI										
Procedural clarity	4.3	(0.6)	2.4	2.4	**95.2**					
Correctness	4.1	(0.7)	4.8	4.8	**90.5**					
Completeness	3.4	(0.9)	**21.4**	26.2	52.4	4.9	(0.5)	3.0	0.0	**97.0**
Complexity	4.2	(0.7)	0.0	11.9	**88.1**	2.2	(1.5)	**66.7**	15.2	18.2
Congruence with current method	3.8	(0.8)	7.1	19.0	73.8	4.3	(1.1)	9.1	12.1	78.8
Observability	3.5	(1.0)	16.7	33.3	50.0	4.3	(1.4)	12.1	6.1	**81.8**
Relevance for client	3.7	(0.7)	2.4	38.1	59.5	3.7	(1.5)	**30.3**	9.1	60.6
Determinants of HCPs/patients										
Personal benefits	3.7	(0.8)	14.3	2.4	**83.3**	3.4	(0.8)	**27.3**	6.1	66.7
Personal disadvantages	3.6	(1.0)	**23.8**	9.5	66.7	3.3	(0.9)	**30.3**	18.2	51.5
Outcome expectations	4.1	(0.5)	2.4	0.0	**97.6**	3.5	(0.9)	**24.2**	6.1	69.7
Task perception	4.4	(0.7)	2.4	4.8	**92.9**	4.4	(1.0)	6.1	12.1	**81.8**
Client satisfaction	3.4	(0.9)	16.7	38.1	45.2	4.6	(1.1)	6.1	6.1	**87.9**
Client cooperation / nurse cooperation	3.5	(0.9)	14.3	28.6	57.1	4.7	(0.9)	9.1	0.0	**90.9**
Social support	3.6	(0.7)	14.3	2.4	**83.3**	3.9	(0.7)	12.1	3.0	**84.8**
Descriptive norm (1–7)^a^	5.5	(1.0)	2.4	16.7	**81.0**	5.2	(1.0)	6.1	15.2	78.8
Subjective norm	3.9	(0.4)	0.0	2.4	**97.6**	3.9	(0.7)	12.1	9.1	78.8
Self-efficacy	4.1	(0.5)	0.0	0.0	**100.0**					
Knowledge	4.0	(0.8)	7.1	9.5	**83.3**					
Awareness of the content of the treatment (1–4)^**b**^	3.5	(0.7)	0.0	0.0	**100.0**	2.9	(1.0)	**57.6**	–	42.4
Determinants of the organisation										
Formal ratification by management (no/yes)			**28.6**	–	71.4					
Replacement when staff leave	2.8	(0.9)	**31.0**	45.2	23.8					
Staff capacity	2.9	(0.9)	**42.9**	23.8	33.3					
Financial resources	2.3	(0.9)	**73.8**	16.7	9.5					
Time available	3.1	(0.8)	**28.6**	35.7	35.7					
Materials, resources and facilities	2.8	(0.9)	**40.5**	33.3	26.2					
Coordinator (no/yes)			**35.7**	–	64.3					
Organisational changes	2.2	(0.8)	**88.1**	2.4	9.5					
Information accessible about the use of the innovation	3.6	(0.9)	9.5	35.7	54.8					
Performance feedback	3.3	(1.0)	**31.4**	35.7	42.9					

*Note.* Scores could range from 1 to 5, unless noted otherwise in parentheses, and higher mean scores reflect a more positive contribution to the implementation of MULTI. Full questions can be found in Additional file 1 (HCPs) and Additional file 2 (patients). HCPs: Healthcare Professionals; neg. = negative response (score < 3); neu. = neutral response (score 3); pos. = positive response (score > 3). Reported barriers (≥ 20% negative response) and facilitators (≥ 80% positive response) are shown in bold

^a^for percentages, calculated to negative (1–3), neutral (4) and positive (5–7)

^b^for percentages, calculated to negative (1–2) and positive (3–4)

Of the implementation determinants related to the HCPs themselves, personal disadvantages were identified as a barrier (24% responded negatively), mainly regarding *the time needed to get patients involved in their day-to-day program*, which was endorsed by all who responded negatively. Among patients, personal benefits and disadvantages were barriers (27 and 30% respectively responded negatively), mostly by disagreeing with the statements *I feel more actively involved in my treatment* (24%), *there is a better atmosphere at the ward* and *I feel better* (both 21%) and agree that participating in MULTI takes a lot of time and energy (both 30%). Outcome expectations were identified as a barrier (24% responded negatively), whereby patients especially disagreed with expecting *more fun and a brighter future* (21%) and *less psychiatric problems* and *more contact with other people* (both 24%). Finally, slightly more than half of the patients reported not to be aware of the content of MULTI.

Implementation determinants related to the organisation were scored low by HCPs (*M* = 2.8, *SD* = 0.5). Except for access to information about the use of MULTI, all determinants related to the organisation were identified as a barrier, with the largest proportions of negative response by HCPs to organisational changes (88%) and financial resources (74%).

Information derived from open-ended questions is shown in Table 3. The topics mentioned most often as barriers by HCPs, were the lack of time for personal development within MULTI (21%), the decrease of support by allied health professionals (20%) and the fact that it takes significant energy to get everyone involved (17%). Patients’ perceived barriers were mixed, with the lack of time to choose their own activities within the group approach most often cited (24%).

**Table 3:** Topics mentioned ≥2x in open-ended questions

	Barriers	*n* (%)	Facilitators	*n* (%)
HCPs (*N* = 42)	Personal development is no specific part of MULTI, which causes a lack of time to support this and to tailor towards patients’ needs^a^	9 (21)	Time for own lifestyle behaviour	4 (9)
	The decrease of support by allied health professionals such as activity coordinators and dietitian due to budget cuts	8 (20)	Better relationship with patients	2 (5)
	It takes a lot of energy to get everyone involved appropriately^b^	7 (17)		
	The shop and restaurant at the hospital where patients can easily buy unhealthy food and beverages – the lack of affordable healthy alternatives	3 (7)		
	Difficult to communicate with patients who do not see or understand the topic of poor physical health	3 (7)		
	Lack of education and clear communication to take away ambiguity and face challenges^c^	3 (7)		
Patients (*N* = 33)	Lack of time within the day-to-day program to choose own activities independent from the group	8 (24)	Activities	17 (52)
	Lack of sports activities	3 (9)	Interaction with peers during activities	3 (9)
	Too much sports activities	3 (9)	Healthier food	3 (9)
	Lack of care after moving to another ward/facility	2 (6)	Daily structure with regular circadian rhythm	2 (6)
			Commitment and support of activity coordinators and nurses during activities	2 (6)

HCPs: Healthcare Professionals

^a^e.g. to find activities meeting patients’ abilities and interests and supporting independence, for example, to prepare them for maintaining an improved lifestyle after moving to another ward/facility. Relapse in both physical and mental health in some patients after moving to other wards or facilities such as sheltered housing

^b^e.g. due to heterogeneity in patients at wards, patients who are unresponsive to motivational interviewing or a lack of consistent action within the team

^c^e.g. dealing with patients who are unresponsive to motivational interviewing and food issues such as binge eating

### Facilitators

Overall, both HCPs and patients responded positively towards implementation determinants related to MULTI (*M* = 3.9, *SD* = 0.6 and *M* = 3.9, *SD* = 0.7, respectively). HCPs responded that the activities required to apply MULTI as intended were clear (95%), not too complicated (88%) and that MULTI was based on factually correct knowledge (91%). For patients, MULTI provided sufficient information about the different possibilities for regular physical activity and healthy nutrition (completeness; 97% responded positively) and outcomes were clearly observable (e.g. effects of MULTI were visible; 82% responded positively).

Also, for implementation determinants related to themselves, both HCPs and patients responded positively overall (*M* = 3.9, *SD* = 0.4 and *M* = 3.8, *SD* = 0.6, respectively). The vast majority of the determinants related to HCPs themselves facilitated the implementation of MULTI. Regarding personal benefits, many HCPs agreed on the statements that with MULTI *work is more fun* (79%), *work is more efficient due to more structure and moments with the group* (71%), there was an *improved relationship with patients* (60%) and there was *less turmoil and incidents at the ward* (55%). Almost all HCPs (98%) agreed on the importance and expectation that MULTI contributed to the intended outcomes (see Additional file 1: Table S1), felt responsible (task perception; 93%), and were positive about subjective norms (98%) and knowledge (83%). They all felt able to put described activities into practice (self-efficacy) and were aware of the content of the treatment. Patients agreed that it is part of their treatment to improve lifestyle (task perception; 82%) and were satisfied with MULTI (client satisfaction; 88%) and nurse cooperation (91%). They responded positively to the availability of adequate assistance within MULTI (social support; 85%), with the most positive response for support by nurses (97%), activity coordinators and dietitian (both 76%) and the least by peers (36%). Implementation determinants related to the organisation showed no facilitators.

Additional information derived out of open-ended questions (Table 3) revealed that the activities were the largest facilitator, as endorsed by 52% of patients.

## Discussion

This is the first study analysing the implementation of a pragmatic lifestyle intervention targeting inpatients with SMI in routine clinical care, including perspectives of both HCPs and patients, as well as the environmental context. In the implementation of MULTI, almost all organisational factors were identified as barriers by HCPs. The strongest barrier in patients was the complexity of participating in MULTI. Many determinants related to MULTI and the HCPs/patients themselves were reviewed as facilitators, reflecting positive attitudes towards such a multidisciplinary integrated approach and their role in it.

### Barriers

Results show that it was complicated for patients to participate in MULTI (complexity). They identified a lack of time within the day-to-day program to choose their own activities, for instance. The results indicate mixed experiences with the day-to-day program as there were positive responses as well regarding barriers such as personal benefits and disadvantages and outcome expectations. This seems to correspond to the lack of time to spend on personal development and tailoring mentioned by HCPs and the time and energy it takes to get patients engaged. Those findings are in line with the previously found contradiction between perceived benefits and barriers in patients with SMI [39], and that it is more difficult to implement lifestyle focused interventions in this population due to challenges they face. These include negative symptoms (e.g. blunted affect, lack of initiative and apathy), cognitive deficits (e.g. memory and attention) [53, 70–73] and low literacy rates [74]. As it is likely that both HCPs and patients were more confronted with those challenges, this may have contributed to perceived barriers related to MULTI and themselves, including the lack of awareness of specific content of MULTI. In line with these results, recent studies suggest that there is both a need to invest more time and emphasis to overcome those challenges by HCPs [75], as well as a need to tailor interventions to address patients’ needs and provide individually meaningful and suitable opportunities to be physically active, which may increase autonomous motivation for those activities [43, 53–55, 59, 76].

However, HCPs mainly reported organisational barriers, such as organisational changes, lack of resources (e.g. financially, replacement and capacity of staff, materials and time), education and decreased support from allied health professionals, which seems to hinder them from making the investment in time and emphasis to overcome aforementioned challenges. Previous studies reported such issues as factors that can negatively impact the support of lifestyle-related behaviour by mental health nurses [43, 77, 78]. Although the ‘change from within’ approach using current staff and resources seemed to be good from the start (overall sustained ownership and commitment), cuts in general budgets and staffing within the hospital – which happened twice during these 18 months – most likely affected MULTI. The reported ambiguity and lack of consistent action within the team seem to correspond with the lack of coordination from the organisation (ambiguity about a coordinator, formal ratification by management, easy access to unhealthy food and beverages within the hospital and lack of performance feedback). A previous study also reported that a lack of coordination and support from management can be a major barrier to successful implementation [45]. Formal ratification by leaders in mental healthcare organisations and identifying a champion who promotes the implementation process are essential [66, 79]. Nevertheless, the fact that we did not find any facilitators in the organisational context may be a result of the ‘bottom-up’ approach. In this implementation at a limited scale within a large mental healthcare organisation – MULTI was developed and carried out at team level without significant involvement of higher management. Therefore, it makes sense that most improvements are needed in organisational determinants, which could contribute to improving tailoring.

### Facilitators

In general, both HCPs and patients were committed towards the design of MULTI as a multicomponent, integrated treatment method, which was emphasised by the response to the open-ended questions. Both agreed that improving lifestyle should be a part of the treatment (task perception) and felt supported by the multidisciplinary team. This confirms the success of a multidisciplinary approach and social support as essential elements in improving lifestyle [51–54, 56, 80–84]. Patients felt especially supported by nurses. Apart from the fact that nurses constitute the largest group of the multidisciplinary team, who have a well-established relationship with patients and regular face-to-face contact [85], this is in line with the design of MULTI whereby nurses participated in activities. The overall perceived social support and interaction, which is also reflected in some open-ended questions and in HCPs indicating less turmoil and incidents and improved relationships with patients, may have contributed to improved social functioning as a result of MULTI [61]. Moreover, our findings seem to confirm previous studies that it is more likely that the lifestyle of patients improves when HCPs are engaged in a healthy lifestyle themselves [75, 80, 86, 87]. The positive response of HCPs towards personal benefits (e.g. work is more fun and better for their lifestyle) and outcome expectations – contrary to patients – may contribute to this engagement. This is consistent with the suggestion that mental health nurses may be more optimistic about physical activity participation than patients [83]. In addition, the participation of HCPs could have a positive ‘side-effect’ as it potentially addresses their high percentage of sedentary behaviour as well [8].

### Limitations and strengths

The results of this study should be considered in light of several limitations. Firstly, excluded patients could have experienced other barriers and facilitators towards participating in lifestyle interventions as they were older and had a higher illness severity. However, including patients with SMI in self-reports or semi-structured interviews remains challenging, given the aforementioned psychopathology and cognitive deficits [53, 70–73]. For future research, with more participants, it would be clinically relevant to study whether patient and disease characteristics predict specific implementation barriers and facilitators. Secondly, with respect to the MIDI questionnaire, the internal consistency for MULTI determinants was lower in patients’ than in HCPs. This may be due to the fact that the questionnaire was not designed for this population. Changes we have made in the MIDI questionnaire to adjust it to our study population (e.g. excluding several items because they were less relevant for patients) could have affected the psychometric properties of the questionnaire as well. Additionally, although the MIDI questionnaire guides HCPs through important organisational determinants, they could have missed other potential barriers and facilitators, as they do not work actively at higher organisational levels in the organisation. Therefore, it might be of value to include higher management as well in the evaluation of barriers and facilitators once they are actively involved in the implementation of such interventions. Nevertheless, the perspective of HCPs on organisational barriers and facilitators is most important in this study, as they play a crucial role in implementing and executing MULTI in daily clinical healthcare. Thirdly, the cut-offs to qualify implementation determinants as barriers (20%) or facilitators (80%) are yet still a rule of thumb and reduced the variable to a dichotomous one. Therefore, it must be noted that items that did not qualify as a barrier or facilitator could still have had a significant influence on the implementation of MULTI. Finally, despite the fact that it plays a major role in the physical health conditions of people with SMI, smoking was not a specific topic in MULTI. As part of an unhealthy lifestyle, smoking is historically and culturally embedded profoundly within inpatient mental health settings [88]. Within clinical practice, many HCPs report barriers to and show negative attitudes towards smoking cessation interventions [89]. This culture and its attendant barriers may have also contributed to the fact that smoking was not a specific topic in MULTI. However, the feasibility of smoking reduction in the longer term was shown in both outpatients and inpatients with SMI [15, 16, 90], despite negative preconceptions and stereotypes. Because of the substantial health benefits, it is advisable to include smoking cessation interventions in the MULTI approach, as well.

A strength of this study is that it includes both the HCPS and the patients’ perspective. To the best of our knowledge, it is the first study analysing the implementation of a lifestyle enhancing intervention in routine clinical care for SMI inpatients. It adds to the limited literature on implementation-related factors, which aim to better understand and eventually close the gap in translating evidence-based interventions into practice. Regarding measurements, social desirability was prevented as much as possible by assessing MIDI anonymously online in HCPs and by working with an independent research assistant to conduct all patient interviews. Furthermore, the MULTI study was conducted in a naturalistic setting (e.g. including all patients and staff without selecting on health status or motivation). This makes it highly relevant for clinical practice, as despite the methodological limitations of the observational design compared to randomised controlled trials, the latter are unlikely to represent both the population (HCPs and patients) as well as organisational factors (e.g. resourcing) under real-world conditions [25]. The response of HCPs was high (75%), which increases the generalizability. This study answers the call for more research to identify and manage barriers and facilitators of implementing lifestyle-related interventions and to understand how these interventions can be delivered successfully in real-world settings, including associated issues within daily mental healthcare [29]. Moreover, it complements a comprehensive evaluation of MULTI, because analyses of the implementation can be used to better interpret the positive changes in health [60, 61] and medication use [62] found in previous studies.

### Implications for clinical practice: directions to address barriers

In clinical practice, HCPs often may be too pessimistic about the ability of people with SMI to embrace health behaviour change, the capabilities of HCPs and the feasibility of change [25, 91]. Our results show that MULTI was feasible in interrupting the general status quo of poor health in inpatients, leading to improvements in a variety of health outcomes [60–62]. Findings show that not only HCPs but also patients are positive towards such an integrated, multidisciplinary, and structured approach, including their own role in it. This emphasises the importance that perceived barriers by management or HCPs should not limit access to the benefits of lifestyle interventions for people living with SMI [91]. We believe that this commitment is largely the result of ownership and multidisciplinary collaboration, whereby HCPs themselves developed a day-to-day program within the clinical context of their wards.

To further improve and disseminate MULTI, action is needed. Recently, a guide was developed to increase the likelihood of successful implementation and scale-up of physical activity interventions in real-world settings and can be used in other areas of public health prevention as well [38]. It describes four iterative steps to 1) characterize the parameters of the implementation setting, 2) identify and engage key stakeholders across multiple levels within the delivery system(s), 3) identify contextual barriers and facilitators to implementation, and 4) address potential barriers to effective implementation. The current study primarily focused on the third step, after step one and two have been part of developing and implementing MULTI. However, reviewing step one and two to address the multiple organisational barriers is needed for further improvement and scale-up of MULTI. This comprises reviewing the key stakeholders (e.g. engaging higher management in addition to the current team) and characterizing implementation setting parameters together. The latter includes the identification of individual or organisational champions and questions like how HCPs can be engaged, trained and supported, how associated costs and resources will be sustainably funded and how implementation processes will be integrated into organisational policies and job descriptions [38]. Together with conveying a vision that inspires HCPs, addressing those topics gives further direction for future work. This is relevant as when it comes to discussions about (dealing with) the availability of particular food and beverages for patients within the hospital, as reported by HCPs. Reviewing these steps will give input to address many organisational barriers found (step 4) and creates a great foundation for further improvement. In the context of education and support, allied health professionals such as activity coordinators and dieticians are essential [54]. They are specialised in addressing specific challenges in physical activity and nutrition, can support mental health staff and contribute to education. Also, setting up community or university partnerships could potentially help with upskilling and enhancing the capabilities of new HCPs [54, 92]. In line with this, knowledge about lifestyle as a topic in mental healthcare must be a part of the curriculum for nurses and other related disciplines.

If such improvements in organisational context can be made, it is expected this would enhance a more unambiguous approach and facilitate HCPs and patients in improving individual tailoring. For this, patients could be more involved in designing the day-to-day program to better target activities towards their abilities, interests and needs, which potentially increases intrinsic motivation. In counselling and informing patients (e.g. psycho education or designing the day-to-day program), tailoring could be improved by taking cognitive challenges faced by people with SMI into account. To enhance retention and comprehension, the use of simplified language and visual materials should be preferred, as well as the use of techniques such as lesson repetition, reading aloud and educational games [53, 93]. Addressing those barriers in implementation and tailoring would largely improve the continuation and further implementation of MULTI, as other implementation determinants are positive. Nevertheless, to confirm current findings and to explore whether the aforementioned suggested improvements are effective, the implementation of MULTI should be studied in different contexts, using the current results and lessons learned. Also, although we know there were little additional costs given the ‘change from within’ approach, there is a need for cost-effectiveness analysis regarding lifestyle interventions [18, 87]. This would especially be of value in the context of considering investments to address barriers.

## Conclusions

To the best of our knowledge, this is the first study analysing the implementation of a lifestyle enhancing treatment within routine clinical care for inpatients with SMI. Findings show important implementation barriers related to organisational factors. Many implementation facilitators related to MULTI and the HCPs/patients themselves reflected positive attitudes towards such a multidisciplinary integrated approach and their own role in it, having driven the implementation of MULTI. We suggest that organisational strategies are needed to further improve and maintain MULTI, including increased management involvement. To confirm and complement current findings, we encourage further implementation and pragmatic research regarding optimal delivery of lifestyle interventions as an integrated component of daily life at inpatient wards.

## Supplementary information

**Additional file 1 Table S1:** Questions used to measure each determinant in healthcare professionals, number of questions and ranges.

**Additional file 2 Table S2:** Questions used to measure each determinant in patients, number of questions and ranges.

## Data Availability

The data that support the findings of this study are not publicly available, because that is inconsistent with the informed consent. However, data are available from the corresponding author on reasonable request and with permission of GGz Centraal.

## Abbreviations

HCPs: Healthcare professionals
MIDI: Measurement instrument for determinants of innovations
MULTI: Multidisciplinary lifestyle enhancing treatment for inpatients with severe mental illness
SMI: Severe mental illness
