# Supplementary material for: Implementation barriers and facilitators of an integrated multidisciplinary lifestyle enhancing treatment for inpatients with severe mental illness: the MULTI study IV
Source: BMC Health Serv Res. 2019 Oct 22;19:740. doi: 10.1186/s12913-019-4608-x (PMC6806487; doi:10.1186/s12913-019-4608-x)
Supplement: Supplementary file 1 — Additional file 1 Table S1: Questions used to measure each determinant in healthcare professionals, number of questions and ranges. [file 12913_2019_4608_MOESM1_ESM.docx]

| **Additional file 1: Table S1** Questions used to measure each determinant in healthcare professionals, number of questions and ranges | | | |
| --- | --- | --- | --- |
| Determinant | Question(s) used to measure determinant | *N* | Range |
| **Determinants of MULTI** | |  |  |
| 1. Procedural clarity | It’s clear to me which activities I should perform within MULTI | 1 | 1-5 |
| 2. Correctness | MULTI is based on factually correct knowledge | 1 | 1-5 |
| 3. Completeness | MULTI provides all the information and materials needed to work with it properly | 1 | 1-5 |
| 4. Complexity | MULTI is too complicated for me to use | 1 | 1-5 |
| 5. Congruence with the current method | MULTI is a good match for how I am used to work | 1 | 1-5 |
| 6. Observability | The outcomes of using MULTI are clearly observable | 1 | 1-5 |
| 7. Relevance for client | I think that MULTI is relevant for our patients | 1 | 1-5 |
|  |  |  |  |
| **Determinants of the user** | |  |  |
| 8. Personal benefits | To what extent does the use of MULTI offer you the following personal advantages?   - Somatic care takes less time - Work is more efficient due to more structure and moments with the group - Improved relationship with patients - Less turmoil and incidents at the ward - Work is more fun | 5 | 1-5 |
| 9. Personal disadvantages | To what extent does the use of MULTI offer you the following personal disadvantages?   - Costs much time to get patients involved in their day to day program - Lack of time for things I have to do as well if I perform MULTI - It forces me to a method that I don’t feel comfortable with - More work-related stress | 4 | 1-5 |
| 10a. Outcome expectations (Importance) | I *find* it important to work in accordance with MULTI to contribute to the following:   - Patients improve:   - Physical activity   - Dietary habits   - Circadian rhythm   - Fun and perspective in life   - Social interaction   - Self-reliance - Better diagnosis and treatment of sleep apnoea - Extensive screening at admission - Reviewing treatment plan within six weeks - Create a multidisciplinary treatment plan in which the patient is involved - When there is a possibility for a patient to move to less intensive care, an evaluation takes place, including (help with) transition to the next facility | 22 | 1-5 |
| 10b. Outcome expectations (Probability) | I *expect* that working in accordance with MULTI contributes to the following:   - Patients improve:   - Physical activity   - Dietary habits   - Circadian rhythm   - Fun and perspective in life   - Social interaction   - Self-reliance - Better diagnosis and treatment of sleep apnoea - Extensive screening at admission - Reviewing treatment plan within six weeks - Create a multidisciplinary treatment plan in which the patient is involved - When there is a possibility for a patient to move to less intensive care, an evaluation takes place, including (help with) transition to the next facility |  |  |
| 11. Task perception | I find working in accordance with MULTI my responsibility | 1 | 1-5 |
| 12. Client satisfaction | Patients are generally satisfied with MULTI | 1 | 1-5 |
| 13. Client cooperation | Patients generally cooperate when I use MULTI | 1 | 1-5 |
| 14. Social support | I can count on adequate assistance (if needed) to use MULTI from….   - Nurses - Nurse practitioner - Psychiatrist - Activity coordinators   - Exercise   - Cooking class and other activities - Dietitian - Volunteers - Patients’ family - Managers | 9 | 1-5 |
| 15. Descriptive norm | In your opinion, what proportion of the colleagues in your organisation for whom MULTI is intended actually use MULTI? | 1 | 1-7 |
| 16a. Subjective norm (Normative beliefs) | Do the following people expect you to use MULTI?   - Client council - Nurses - Nurse practitioner - Psychiatrist - Activity coordinators   - Exercise   - Cooking class and other activities - Dietitian - Volunteers - Patients’ family - Managers | 20 | 1-5 |
| 16b. Subjective norms (Motivation to comply) | When it comes to working in accordance with MULTI, to what extent do you comply with the opinions of the following people?   - Client council - Nurses - Nurse practitioner - Psychiatrist - Activity coordinators   - Exercise   - Cooking class and other activities - Dietitian - Volunteers - Patients’ family - Managers |  |  |
| 17. Self-efficacy | Should you wish to do so, do you think you can put the activities described below into practice?   - Stimulate patients to actively participate in the daily structure at the ward - Create a tailored day-to-day activity program together with the patient - Align these programs and activities with other disciplines - Improve patients’ dietary habits - Offer patients a tailored physical activity program - Coach a patient how to maintain a healthier lifestyle if he/she moves to less intensive healthcare or another ward - Stimulate and inspire your colleagues to actively contribute to and participate in MULTI - Use MULTI as general policy within long-term mental healthcare | 8 | 1-5 |
| 18. Knowledge | I have enough knowledge to be able to use MULTI | 1 | 1-5 |
| 19. Awareness of contents of MULTI | To what extent are you informed about the content of MULTI? | 1 | 1-4 |
| **Determinants of the organisation** | |  |  |
| 20. Formal ratification by management | Formal agreements have been made by management about the use of MULTI within our organisation | 1 | Yes/no |
| 21. Replacement when staff leave | In my organisation, there are arrangements in place so that staff who uses the innovation but leaves the organisation are replaced in good time | 1 | 1-5 |
| 22. Staff capacity | There are enough people in our organisation to use MULTI as intended | 1 | 1-5 |
| 23. Financial resources | There are enough financial resources available to use MULTI as intended | 1 | 1-5 |
| 24. Time available | There is enough time available to be able to work in accordance with MULTI | 1 | 1-5 |
| 25. Material resources and facilities | Our organisation provides me with enough materials and other resources or facilities necessary to be able to work according to MULTI as intended ^a^ | 1 | 1-5 |
| 26. Coordinator | Within our organisation, at least one person has been appointed to coordinate the implementation of MULTI | 1 | Yes/no |
| 27. Organisational changes | To what extent do organisational changes impede the implementation of MULTI?   - Relocations of patients - Cuts (less money or staff) - Lack of integrated/permanent colleagues in the team | 3 | 1-5 |
| 28. Information accessible about the use of MULTI | I have easy access to information about the use of MULTI |  |  |
| 29. Performance feedback | In my organisation, feedback is regularly provided about the progress of the implementation of MULTI | 1 | 1-5 |
|  | | | |
