# Supplementary material for: Implementation barriers and facilitators of an integrated multidisciplinary lifestyle enhancing treatment for inpatients with severe mental illness: the MULTI study IV
Source: BMC Health Serv Res. 2019 Oct 22;19:740. doi: 10.1186/s12913-019-4608-x (PMC6806487; doi:10.1186/s12913-019-4608-x)
Supplement: Supplementary file 2 — Additional file 2 Table S2: Questions used to measure each determinant in patients, number of questions and ranges. [file 12913_2019_4608_MOESM2_ESM.docx]

| **Table S2** Questions used to measure each determinant in patients, number of questions and ranges | | | |
| --- | --- | --- | --- |
| Determinant | Question(s) used to measure determinant | *N* | Range |
| **Determinants of MULTI** | |  |  |
| 1. Completeness | There is sufficient information available about the different possibilities I have for regular physical activity and healthy nutrition | 1 | 1-5 |
| 2. Complexity | It is too complicated for me to participate in MULTI | 1 | 1-5 |
| 3. Congruence with the current method | The lifestyle treatment fits well with how we aim to work at the ward | 1 | 1-5 |
| 4. Observability | The outcomes of MULTI are clearly observable | 1 | 1-5 |
| 5. Relevance for client | I think that MULTI is relevant for me | 1 | 1-5 |
|  |  |  |  |
| **Determinants of the user** | |  |  |
| 6. Personal benefits | To what extent does the use of MULTI offer you the following personal advantages?   - I have less physical issues - I do more in one day than I did before - I feel more actively involved in my treatment - There is a better atmosphere at the ward - I feel better | 5 | 1-5 |
| 7. Personal disadvantages | To what extent does the use of MULTI offer you the following personal disadvantages?   - Participating in MULTI takes a lot of time - Participating in MULTI takes a lot of energy - MULTI forces me to a way of living that I am not comfortable with - MULTI causes (extra) stress | 4 | 1-5 |
| 8. Outcome expectations | Do you expect the following things to happen due to MULTI:   - I am more physically active - I have healthier dietary habits - I have less psychic problems - My daily structure improves (more regular circadian rhythm) - I have more fun and a brighter future - I am more in contact with other people - I can do more myself | 7 | 1-5 |
| 9. Task perception | I think it is part of my treatment to improve my lifestyle | 1 | 1-5 |
| 10. Client satisfaction | I am satisfied with MULTI | 1 | 1-5 |
| 11. Nurse cooperation | The nurses implemented MULTI very well | 1 | 1-5 |
| 12. Social support | I can count on adequate assistance (if needed) to use MULTI from….   - Nurses - Psychiatrist - Activity coordinators   - Exercise   - Cooking class and other activities - Dietitian - Peers | 6 | 1-5 |
| 13. Descriptive norm | In your opinion, what proportion of the team for whom MULTI is intended actually uses MULTI? | 1 | 1-7 |
| 14. Subjective norm | Do the following people expect you to use MULTI?   - Nurses - Psychiatrist - Activity coordinators   - Exercise   - Cooking class and other activities - Dietitian - Peers | 6 | 1-5 |
| 15. Awareness of contents of MULTI | To what extent are you informed about the content of MULTI? | 1 | 1-4 |
|  | | | |
